# Willingness to Participate in Medical Research Among Adults in the United Arab Emirates (UAE): A Cross-Sectional Study

**DOI:** 10.7759/cureus.81894

**Published:** 2025-04-08

**Authors:** Lina Hajjar, Sumayah Al Khojah, Mukram A Jamour, Abdullah M Hajjo, Ghina O Fansa, Amal Hussein, Rania S Ahmed

**Affiliations:** 1 College of Medicine, University of Sharjah, Sharjah, ARE; 2 Family and Community Medicine, University of Sharjah, Sharjah, ARE

**Keywords:** consent, expectations, medical research, participation, public knowledge, recruitment, united arab emirates (uae)

## Abstract

Introduction: Medical research plays a major role in advancing healthcare, yet public participation remains critical for its success. This study aims to evaluate the willingness to participate among adults in the United Arab Emirates (UAE), investigate the factors that influence their choices, raise their expectations, and level of awareness about medical research.

Methods: A cross-sectional study conducted between February and March of 2022 included 350 adult UAE residents. The study used a self-administered online questionnaire distributed across known social media platforms (WhatsApp, Instagram, Facebook). As part of the study's methodology, participants were categorized according to their socioeconomic status, demographics, and history of involvement in research. Statistical analysis was performed to identify significant factors affecting willingness to participate.

Results: Age, gender, and marital status did not significantly affect a person's willingness to participate in medical research, but having previously conducted research and trusting medical researchers did. Participants expressed a strong preference for less invasive research, with 277 (79.1%) likely to engage in short questionnaires, while only 94 (26.9%) were willing to participate in studies involving new drugs or procedures. Recruitment methods also played a role, with phone calls being the most effective. Selfless concerns were the primary motives of most participants 156 (49.8%), while health concerns were the main deterrent.

Conclusion: The study shows that trust in researchers, prior experience, and the nature of the research significantly influence participation among UAE residents. These findings emphasise the need for targeted educational initiatives to improve public understanding of medical research and build trust, which could boost participation in essential studies. Incorporating community input into research design and health policies would better align medical innovation with public needs, advancing the UAE's goals of becoming a leader in evidence-based and precision medicine.

## Introduction

From William Withering’s pioneering trial on digitalis to the rapid global vaccine development during the COVID-19 pandemic, medical research has continually redefined itself as a cornerstone of healthcare, driving innovation and enhancing patient outcomes. This historical evolution highlights how ongoing research not only builds on past discoveries but also repeatedly reshapes our understanding and treatment of diseases, with direct implications for patient care and well-being. Despite significant improvements in medical research over the past decade, Arab countries remain behind the global forefront. The United Arab Emirates (UAE), ranked 58th in the world, contributed to 5.1% of medical research output among Arab nations between 2007 and 2016, trailing Egypt (32.1%), Saudi Arabia (28.4%), Tunisia (11.4%), Lebanon (5.9%), and Morocco (5.2%) [1]. Furthermore, recent trends showed a notable increase of 25.2% in health research outputs in the country from 2017 to 2022 [2].

Aligned with UAE Vision 2021, the country has undertaken numerous initiatives to advance medical research, including strategic investments in research infrastructure, fostering international collaborations with leading global institutions, expanding funding opportunities, and developing innovative research initiatives such as the Dubai Health Authority’s Health Innovation Lab [3]. However, these efforts could be undermined without sufficient public participation in research studies. A lack of public engagement in medical research can limit the generalizability and applicability of findings, slowing medical advancements and affecting healthcare policies, clinical guidelines, and treatment options. Understanding the factors influencing participation is essential for improving research inclusivity and ensuring that medical discoveries benefit diverse populations.

In this study, which is considered original in the UAE, our primary objective is to investigate the willingness to participate in medical research among adults in the UAE. Secondary objectives include identifying factors influencing participation, such as demographics, education, employment, research duration, trust in medical staff, and the impact of the COVID-19 pandemic. We also examine public knowledge, awareness of research importance, recruitment methods, and whether compensation affects participation willingness.

## Materials and methods

Study design

This is a cross-sectional, descriptive study conducted among adult UAE residents, defined as individuals aged 18 and above, between February and March 2022. This study design was chosen for its ability to provide a timely, cost-effective snapshot of public attitudes while facilitating data collection from a large, representative sample. It also allows for the analysis of demographic and knowledge-based factors influencing participation. A self-administered online questionnaire was used to accommodate the social distancing and precautionary measures implemented in the UAE (see Appendices). Volunteer sampling (self-selection sampling) was employed, and data were collected through a 39-item questionnaire distributed via multiple social media platforms (WhatsApp, Facebook, Instagram, etc.), available in both Arabic and English. Those social media platforms were selected due to their widespread use in the UAE, enabling broad outreach across different demographic groups. Exclusion criteria included non-residents, individuals under 18 years old, mentally handicapped individuals, and those unable to speak Arabic or English.

The sample size for this study was calculated using the following formula: n=4p(1-p)/SE^2^, where *n* is the sample size, *p* is the expected proportion % (expected prevalence), and *SE* is the sampling error or margin of error. *SE* was set at 0.05, and *p* was set at 0.5. Substituting in the formula yields *n* = 424.

Questionnaire development

The lack of an existing tool to assess attitudes towards participation in medical research among adults in the UAE led us to develop our own self-administered questionnaire. It consisted of four main sections: Demographics, knowledge, expectations, and factors affecting participation in medical research. The questionnaire included five-item Likert scales, yes/no questions, and multiple-choice questions. It was initially written in English, translated into Arabic, and reviewed multiple times to ensure consistency. While it was assessed by a panel of experts in medical research for content validity and underwent a pilot study with a small sample representative of the study population to evaluate clarity and comprehension, the tool was not formally validated. Based on the feedback, necessary modifications were made before finalising the questionnaire for data collection.

Study outcomes

This study is mainly aimed at assessing the willingness of adult UAE residents to participate in medical research, the different factors that may influence their decision to participate or not, their knowledge regarding medical research, and what expectations they may have from participating in it.

Data analysis

Data were analyzed using IBM SPSS Statistics for Windows, Version 28.0 (IBM Corp., Armonk, NY, USA). No missing data was encountered due to the nature of the online questionnaire, where submission required responding to all items. The age groups 50-59 years and 60+ years were combined into one group due to the low number of responses. Marital status was made into two groups: single or married. The city of residence was condensed into four groups, one for each of the most populous emirates (Abu Dhabi, Dubai, and Sharjah) and one for all the other emirates. Monthly household income groups were also made into three groups: less than 10,000 AED (UAE dirham), 10,000-20,000 AED, and more than 20,000 AED.

As all measured variables were categorical, bivariate analysis using the Chi-square test was conducted to identify factors significantly associated with willingness to participate in medical research. The Chi-square test is appropriate for examining relationships between categorical variables such as age group, gender, prior research experience, and trust in researchers. A p-value of less than 0.05 was considered statistically significant. While multivariate analyses like logistic regression could offer deeper insight by adjusting for confounders, this descriptive cross-sectional study focused on exploring associations rather than building predictive models.

This study was reviewed and approved by the Research Ethics Committee of the University of Sharjah (Reference Number: REC-22-02-14-07-S, dated February 14, 2022). It was conducted in accordance with all relevant guidelines and regulations.

## Results

Participants’ profile

A total of 350 participants were included in this study. Female participants comprised 206 (58.9%), and male participants accounted for 142 (40.6%) of the participants. Participants were divided into two main adult age groups, with 226 (64.6%) below 40 and 124 (35.4%) aged 40 years or more. Out of the total sample, 166 (47.4%) had an undergraduate degree, and 170 (48.6%) were employed. Only 135 (38.6%) were studying or working in the medical field. Married participants, 183 (52.3%), formed the majority, while single participants accounted for 167 (47.7%). With regards to suffering from a chronic disease like diabetes, hypertension, or dyslipidemia, 280 (80.0%) reported no chronic illness. On the contrary, 181 (51.7%) have at least one first-degree relative suffering from a chronic disease (Table 1).

**Table 1:** Demographic characteristics of participants AED: United Arab Emirates dirham

Variable	Category	n (%)
Age (years)	18-29	165 (47.1)
	30-39	61 (17.4)
	40-49	60 (17.1)
	50+	64 (18.3)
Gender	Male	142 (40.6)
	Female	206 (58.9)
	Prefer not to say	2 (0.60)
Marital Status	Single	167 (47.7)
	Married	183 (52.3)
City of residence	Abu Dhabi	88 (25.1)
	Dubai	102 (29.1)
	Sharjah	128 (36.6)
	Other	32 (9.1)
Ethnicity	Local	43 (12.3)
	Arab non-local	275 (78.6)
	Non-Arab	32 (9.1)
Employment status	Student	122 (34.9)
	Employed	170 (48.6)
	Unemployed	58 (16.6)
Educational level	High school or less	75 (21.4)
	Diploma	40 (11.4)
	Undergraduate degree	166 (47.4)
	Post-graduate degree	69 (19.7)
Monthly income (AED)	Less than 10,000	111 (31.7)
	10,000-20,000	106 (30.3)
	More than 20,000	133 (38.0)
History of chronic disease	Yes	70 (20.0)
	No	280 (80.0)
1st degree relative with history of chronic disease	Yes	181 (51.7)
	No	169 (48.3)
Working/studying in medical field	Yes	135 (38.6)
	No	215 (61.4)
Prior participation in medical research	Yes	82 (24.3)
	No	255 (75.7)

Willingness to participate in medical research

**Figure 1:**
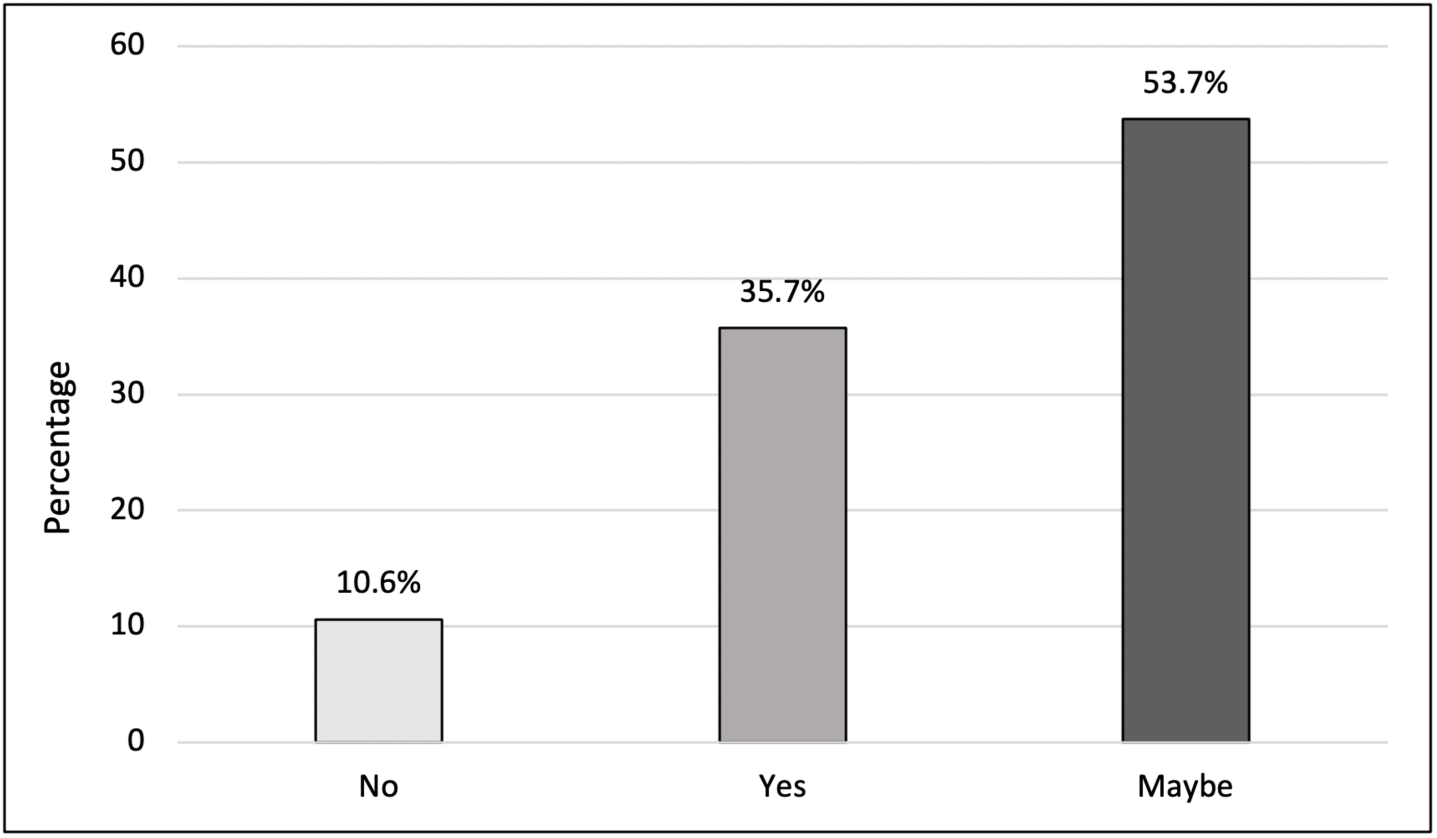
Willingness to participate in medical research

In response to a question about their willingness to participate in medical research, 188 (53.7%) replied "Maybe", while 125 (35.7%) showed a positive attitude and were willing to participate, and 37 (10.6%) had no intention for future participation (Figure 1).

Bivariate analysis revealed that demographic and socio-economic factors, including age, gender, marital status, city of residence, ethnicity, employment status, educational level, monthly income, and working/ studying in the medical field, did not significantly influence willingness to participate in medical research (p >0.05). However, participants with prior participation in medical research 40 (48.8%) were two times more likely to participate (p=0.006) in future studies compared to those with no prior participation (32.2%). In addition, having a chronic disease significantly increased the willingness to participate in medical research requiring follow-up for more than a year by 1.7 fold (p = 0.016).

Although it is not the most preferred method of recruitment, when asked about getting invited through a phone call, participants were 1.7 times more willing to participate in medical research than when using other recruitment methods (p=0.028). Furthermore, trust in medical researchers was significantly associated with increased willingness to participate, whereas those who trusted researchers were 2.9 times more willing to participate (p < 0.001). Interestingly, the COVID-19 pandemic had a profound effect on willingness, making individuals approximately five times more willing to participate in medical research (p < 0.001) than prior to the pandemic.

Preferences of the degree of involvement in medical research

**Figure 2:**
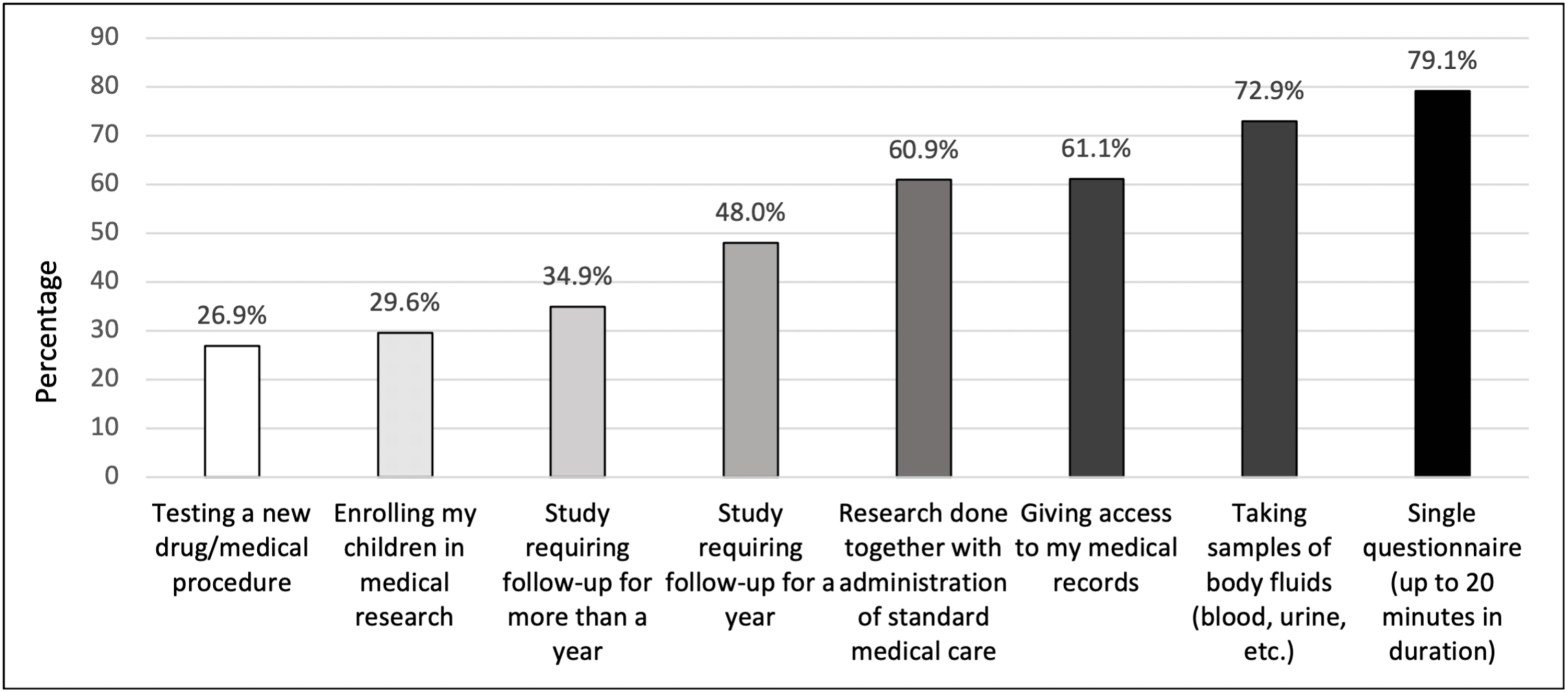
Proportion of subjects who are likely/very likely to participate in medical research

**Figure 3:**
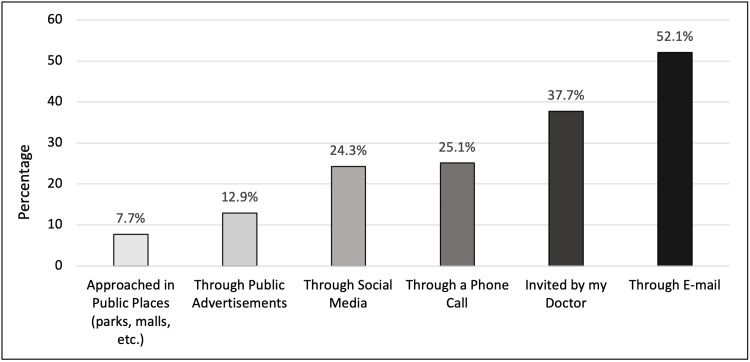
Preferred method of recruitment for medical research

The majority of participants, 277 (79.1%), were either “very likely” or “likely” to participate in medical research involving a single questionnaire lasting up to 20 minutes in duration. Medical research that requires the collection of body fluid samples (like blood or urine) was slightly less preferred with 255 (72.9%) participants either “very likely” or “likely” to participate. Among the participants, 214 (61.1%) were “very likely” or “likely” to participate in research requiring access to medical records. Next preferred was research that involves follow-up for a year with 168 (48%) and follow-up for more than a year with 122 (34.9%) of participants either “very likely” or “likely” to participate. Experimenting with a new drug or medical procedure was least preferred, with only 94 (26.9%) participants either “very likely” or “likely” to participate (Figure 2). The preferred methods of recruitment for medical research are shown in Figure 3.

Knowledge, motives, and concerns for participation

**Figure 4:**
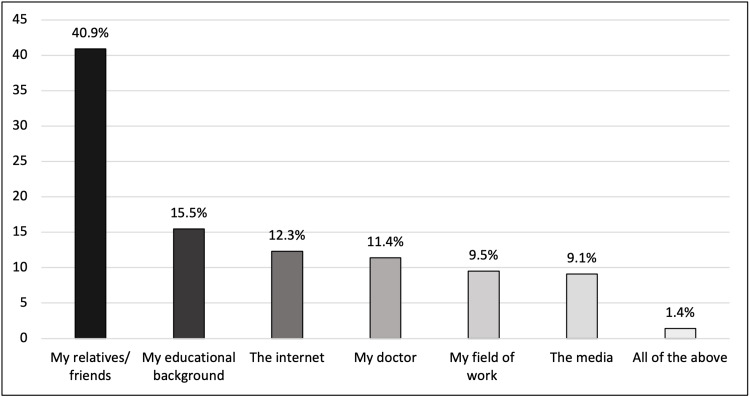
Source of knowledge about medical research

Among the two main divisions of research, 140 (40.0%) of participants had prior knowledge of both observational and experimental studies. Comparing both types, people generally had more prior knowledge of observational studies than experimental studies. Among the participants, 126 (36.0%) had no knowledge of any type of medical research. A greater proportion of participants (40.9%; n=90) obtained their knowledge about medical research from relatives and friends, whereas smaller proportions obtained their knowledge from their educational background, the internet, their doctors, field of work, and the media, respectively (Figure 4). 

**Figure 5:**
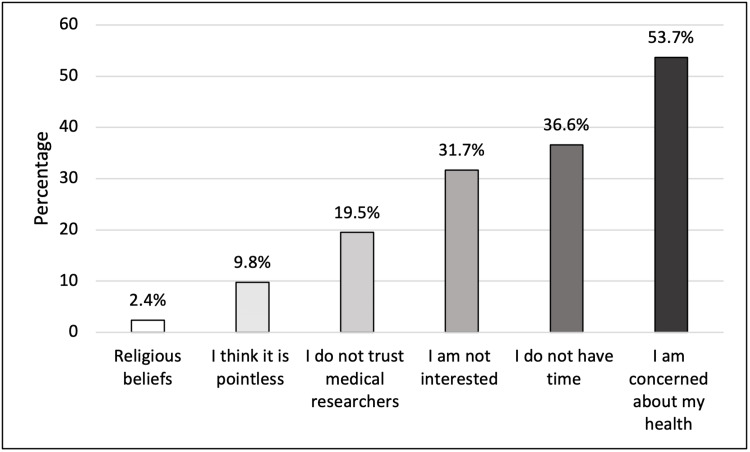
Reasons for refusing to participate in medical research

Out of the total number of participants, 156 (49.8%) had purely altruistic motives for participating in medical research compared to other expected compensations like receiving a certificate, free medical care, cover for expenses, and money. Personal health concerns were the most frequently cited reason for not participating (53.7%), whereas religious beliefs were the least commonly mentioned reason (2.4%) (Figure 5).

## Discussion

The main result answering our primary objective showed that the majority of the adult population in the UAE were uncertain about their future participation in medical research (53.7%). A considerable minority had a positive attitude regarding medical research and were willing to participate (35.7%) purely out of altruistic motives.

This quantifies the degree of support that the general public in the UAE has towards medical research as reflected by their willingness to participate in studies. These results have implications for both public policy and practical applications. From a public policy standpoint, widespread public involvement in medical research is crucial because it encourages evidence-based decision-making. This approach is the primary method used in delivering health services today. It demands that medical and social service providers identify and prove the effectiveness and cost-efficiency of treatments. For evidence-based practices to work, people must be willing to participate in research studies that thoroughly evaluate different treatment methods and identify their weak points. For those conducting medical research, the current study indicates that there are opportunities to boost participation by focusing recruitment efforts not only on those already willing but also on those who are uncertain about participating. This would involve customising communication strategies to address the specific traits and concerns of each group [4]. The Transtheoretical Model and Stages of Change [5], which is an approach used to provoke behavioural changes such as quitting smoking, managing weight, and undergoing cancer screenings [6], could be adapted to improve participation in medical research studies. The findings of this study reflect varying levels of public readiness to engage in healthcare decision-making in the UAE. Many participants were in the pre-contemplation or contemplation stages, showing limited awareness or uncertainty about their role. Some showed interest in becoming more involved, indicating a shift toward preparation, while a smaller group had already taken action. Trust in the healthcare system, cultural norms, and perceived barriers influenced progression through these stages. Applying this model helps identify where the public stands and highlights key areas for targeted interventions to encourage greater and sustained participation. To design effective messages that encourage participation, it is essential to consider the unique characteristics and concerns of both willing and unsure groups.

In general, factors like age, gender, marital status, city of residence, ethnicity, employment status, educational level, and monthly income had no influence on the willingness to participate in medical research. A similar study carried out in Tunisia showed that healthy volunteers, young participants (<40 years), high educational level, presence of chronic disease, and prior history of blood donation were associated with willingness to participate in medical research [7]. Another study in Saudi Arabia on willingness to enroll in phase I of clinical trials demonstrated that younger people are more willing to enroll as compared to older ones (>=30) [8].

In addition, being ill or having a first-degree relative who is ill does not seem to affect an individual’s decision. If anything, this might suggest that the general public does not make a direct connection between participating in medical research and the evolution of novel treatments and therapies that can help with their medical illness [9]. Also, people in this part of the world might have a perceived assumption that new treatments and therapies exclusively emerge from the Western part of the world and that medical research conducted in the Middle East would not have much impact [10].

Working or studying in the medical field does not affect an individual’s willingness to participate. This may be due to the diverse viewpoints people have regarding how medical research is conducted and their differing evaluations of the potential benefits and risks involved. Knowing what could go wrong can cancel out the sense of familiarity that people involved in the medical field have towards medical research [11]. Previous participation in medical research and the level of trust in medical researchers positively influenced individuals' willingness to participate [12]. Such prior involvement may have enhanced respondents' understanding of the diverse range of participants in research studies, making them aware of the inclusion of individuals from various backgrounds, including the wealthy, impoverished, healthy, ill, and terminally ill [4]. Additionally, this experience might have fostered greater trust in medical researchers, creating a positive feedback loop where increased understanding and trust mutually reinforce each other. Although having a medical condition did not significantly affect the overall willingness to participate, there was an unexpectedly large number of people indicating their willingness to participate in medical research that requires follow-up for more than a year. This may be attributed to their desire to access free healthcare through their participation, which translates to significant financial benefit, particularly if it is guaranteed for more than a year [13]. Despite being less preferred than invitations through emails or by their doctors, recruiting individuals via phone calls notably enhanced their willingness to participate. This can be explained by the personal touch of the call, which makes them feel valued, helps dispel concerns of being contacted by spam, and decreases the chance of potential conflicts of interest that might exist when invited through other methods. Interestingly, the COVID-19 pandemic had a significant impact on willingness to participate in medical research, with participants being five times more likely to engage in research after the pandemic. At the time of data collection, the UAE was recovering from the effects of the pandemic, and people were beginning to realise the real-life consequences of unresolved viral disease and how it can directly threaten human life. This experience was an eye-opener to the vital role of medical research in tackling global health crises [14].

Participants preferred engaging in medical research through a single questionnaire, lasting up to 20 minutes, likely due to its greater sense of control, safety, time efficiency, and convenience compared to methods involving body fluid collection, medical record access, or long-term follow-ups [15]. In contrast, participating in clinical trials involving new drugs or medical procedures raises greater risks and concerns about potential adverse effects; thus, it was the least preferred option. Concerns about personal well-being were the most frequently cited reasons for opting out of medical research [16].

Despite ongoing efforts to improve public understanding of medical research and its various types, 36% of participants in the study remain unfamiliar with observational and experimental studies. While it does not represent the majority, this gap in knowledge may reduce willingness to participate.

There were several limitations to our study. First, our sample likely included individuals who are naturally more inclined to participate in medical research than the general population, as it was limited to those who agreed to complete our questionnaire and participate in the study. Second, the study assessed general attitudes toward medical research, which may have led participants to express more enthusiasm than they would in real-life scenarios. Their responses may reflect intentions rather than actual behaviours, and dropout rates in future studies may remain high despite initial agreement, making completion rates uncertain. Third, the survey focused solely on participants' perspectives without accounting for potential influence from family members on their decision-making. Fourth, distributing the questionnaire exclusively through social media platforms limited our reach to diverse demographic groups, unintentionally under-representing older adults and individuals without social media access. Fifth, we did not control for potential confounding variables, as no multivariate analysis such as logistic regression was performed. This may have allowed unmeasured or overlapping variables to influence observed associations, limiting our ability to identify independent predictors of participation. Finally, the assessment of participants’ knowledge about medical research was based on a non-validated tool.

## Conclusions

Most of the participants in this study replied "maybe" to the question of whether they wanted to participate in future medical research, with higher chances of participation associated with invitations through a phone call, building greater trust in medical researchers, and having a prior experience, with special preference to single questionnaires of up to 20 minutes in duration.

Concerns about personal health-related adverse events was the most frequent justification for refusing to participate in future medical research. To build a research-supportive culture, policymakers should invest in public education campaigns, improve communication about study safety, and incorporate community input into research planning. Strengthening trust and accessibility will be essential for aligning the population with the UAE’s vision of becoming a leader in medical innovation and evidence-based healthcare.

## Appendices

**Table 2:** English version of the self-administered online questionnaire UAE: United Arab Emirates, AED: United Arab Emirates dirham

Category	Question	Answer options
Willingness to participate	W. Are you willing to participate in this survey?	Yes
		No, I don't mind saying why (skip to question BR1)
		No, I don't want to say why
Demographic information	D1. What is your gender?	Male
		Female
		Prefer not to say
	D2. Select your age range:	<18
		18-29
		30-39
		40-49
		50-59
		60+
	D3. What is your residential status?	UAE local
		UAE resident
		Visitor
	D4. What is your marital status?	Married
		Widowed
		Divorced
		Single
	D5. What is your city of residence?	Sharjah
		Dubai
		Ajman
		Abu Dhabi
		Ras Al Khaimah
		Umm Al Quwain
		Al-Fujairah
	D6. What is your ethnicity?	Emirati
		Arab (non-Emirati)
		Non-Arab
	D7. What is your employment status?	Student
		Employed and studying
		Employed
		Unemployed
		Housewife
		Retired
		Other (specify)
	D8. What is your highest education level?	High school or less
		Diploma
		Undergraduate degree
		Master’s degree or equivalent
		Doctorate or equivalent
		Other (specify)
	D9. What is your household income per month?	Less than 5,000 AED
		5,000 - 10,000 AED
		10,000 - 15,000 AED
		15,000 - 20,000 AED
		More than 20,000 AED
Medical background	B1. Do you suffer from any long-term medical conditions?	Yes
		No
	B2. Do any of your 1st-degree relatives suffer from a long-term medical condition?	Yes
		No
	B3. Do you work or study in the medical field?	Yes
		No
Knowledge and awareness about medical research	K1. Which type of medical research have you heard about?	Observational studies
		Experimental studies
		Both
		None
	K2. Where did you hear about medical research from?	My doctor
		The media
		The internet
		Relatives/friends
		Educational background
		Field of work
		Other (specify)
	K3. Please indicate your level of agreement with each of the following statements	Likert scale (very likely, likely, neutral, unlikely, very unlikely)
	Confidentiality of research participants is adequately protected in research	Very likely
		Likely
		Neutral
		Unlikely
		Very unlikely
	Medical research benefits society	Very likely
		Likely
		Neutral
		Unlikely
		Very unlikely
	Medical research is an essential step in developing new treatments	Very likely
		Likely
		Neutral
		Unlikely
		Very unlikely
	Participation in research is entirely voluntary	Very likely
		Likely
		Neutral
		Unlikely
		Very unlikely
	Participants can leave the research at any point in time if they feel like it	Very likely
		Likely
		Neutral
		Unlikely
		Very unlikely
	Participation in research causes risk	Very likely
		Likely
		Neutral
		Unlikely
		Very unlikely
Factors affecting participation	F1. Have you ever participated in medical research before?	Yes
		No
	F2. Rate your level of agreement with each of the following statements:	Likert scale (strongly agree, agree, neutral, disagree, strongly disagree)
	I trust medical researchers	Strongly agree
		Agree
		Neutral
		Disagree
		Strongly disagree
	If the research is about a new topic, I am less likely to participate	Strongly agree
		Agree
		Neutral
		Disagree
		Strongly disagree
	The current pandemic has made me less likely to participate in medical research	Strongly agree
		Agree
		Neutral
		Disagree
		Strongly disagree
	The current pandemic has made me more likely to participate in medical research	Strongly agree
		Agree
		Neutral
		Disagree
		Strongly disagree
	F3. If you were invited to participate in medical research, would you be willing to accept?	Yes, for sure
		No (skip to BR1)
		Maybe, it depends
	F4.How likely are you willing to participate in the following:	Likert scale (very likely, likely, neutral, unlikely, very unlikely)
	Single questionnaire (up to 20 minutes in duration)	Very likely
		Likely
		Neutral
		Unlikely
		Very unlikely
	Giving body fluid samples (blood, urine, etc.)	Very likely
		Likely
		Neutral
		Unlikely
		Very unlikely
	One-time visit	Very likely
		Likely
		Neutral
		Unlikely
		Very unlikely
	Study requiring follow-up for a year	Very likely
		Likely
		Neutral
		Unlikely
		Very unlikely
	Study requiring Follow-up for more than a year	Very likely
		Likely
		Neutral
		Unlikely
		Very unlikely
	Research with standard medical care	Very likely
		Likely
		Neutral
		Unlikely
		Very unlikely
	Testing a new drug or medical procedure	Very likely
		Likely
		Neutral
		Unlikely
		Very unlikely
	Giving access to my medical record	Very likely
		Likely
		Neutral
		Unlikely
		Very unlikely
	Enrolling my children in research	Very likely
		Likely
		Neutral
		Unlikely
		Very unlikely
	F5. How would you like to be invited for research participation?	E-mail
		Social media
		Phone call
		Invited by my doctor
		Public advertisements
		Approached in public places (parks, malls, etc.)
		None
		Other (specify)
Expectations from participating in medical research	E1. What do you expect in return for participating in medical research? (Tick all that apply)	Nothing, I just want to help others
		Nothing, I just want to contribute to science
		Money
		Free medical care
		Certificate
		Compensation (travel, parking, etc.)
		Other (specify)
Barriers to participation	BR1. Why would you not participate in medical research?	I do not have time
		I am not interested
		Religious beliefs
		I am concerned about my health
		I think it is pointless
		I do not trust researchers
		Other (specify)
